# An external validation of the Kidney Donor Risk Index in the UK transplant population in the presence of semi-competing events

**DOI:** 10.1186/s41512-023-00159-9

**Published:** 2023-11-21

**Authors:** Stephanie Riley, Kimberly Tam, Wai-Yee Tse, Andrew Connor, Yinghui Wei

**Affiliations:** 1 Centre for Mathematical Sciences, School of Engineering, Computing and Mathematics, University of Plymouth, Plymouth, UK; 2 School of Engineering, Computing and Mathematics, University of Plymouth, Plymouth, UK; 3 Department of Renal Medicine, South West Transplant Centre, University Hospitals Plymouth NHS Trust, Plymouth, UK

**Keywords:** Survival analysis, Time-to-event model, Competing events, Risk prediction, External validation, Kidney transplantation

## Abstract

**Background:**

Transplantation represents the optimal treatment for many patients with end-stage kidney disease. When a donor kidney is available to a waitlisted patient, clinicians responsible for the care of the potential recipient must make the decision to accept or decline the offer based upon complex and variable information about the donor, the recipient and the transplant process. A clinical prediction model may be able to support clinicians in their decision-making. The Kidney Donor Risk Index (KDRI) was developed in the United States to predict graft failure following kidney transplantation. The survival process following transplantation consists of semi-competing events where death precludes graft failure, but not vice-versa.

**Methods:**

We externally validated the KDRI in the UK kidney transplant population and assessed whether validation under a semi-competing risks framework impacted predictive performance. Additionally, we explored whether the KDRI requires updating. We included 20,035 adult recipients of first, deceased donor, single, kidney-only transplants between January 1, 2004, and December 31, 2018, collected by the UK Transplant Registry and held by NHS Blood and Transplant. The outcomes of interest were 1- and 5-year graft failure following transplantation. In light of the semi-competing events, recipient death was handled in two ways: censoring patients at the time of death and modelling death as a competing event. Cox proportional hazard models were used to validate the KDRI when censoring graft failure by death, and cause-specific Cox models were used to account for death as a competing event.

**Results:**

The KDRI underestimated event probabilities for those at higher risk of graft failure. For 5-year graft failure, discrimination was poorer in the semi-competing risks model (0.625, 95% CI 0.611 to 0.640;0.611, 95% CI 0.597 to 0.625), but predictions were more accurate (Brier score 0.117, 95% CI 0.112 to 0.121; 0.114, 95% CI 0.109 to 0.118). Calibration plots were similar regardless of whether the death was modelled as a competing event or not. Updating the KDRI worsened calibration, but marginally improved discrimination.

**Conclusions:**

Predictive performance for 1-year graft failure was similar between death-censored and competing event graft failure, but differences appeared when predicting 5-year graft failure. The updated index did not have superior performance and we conclude that updating the KDRI in the present form is not required.

**Supplementary Information:**

The online version contains supplementary material available at 10.1186/s41512-023-00159-9.

## Introduction

For many patients with end-stage kidney disease, transplantation represents the optimal treatment. The demand for deceased donor kidneys in the United Kingdom (UK) greatly outweighs availability [1]. It is therefore essential to maximise the number of successful transplants in order to reduce the number of recipients returning to the transplant waiting list or dialysis. A prediction model may provide support to clinicians charged with deciding whether to accept the offer of a donor kidney for an individual patient. Such models can incorporate a large number of donor, recipient and transplant-related factors to produce personalised risk predictions.

In the United States (US), the Kidney Donor Risk Index (KDRI), proposed by Rao et al. [2], is used as part of the allocation process for deceased donor kidneys to those awaiting a kidney transplant. It was originally developed to predict graft failure in first-time, kidney-only, adult transplants with the intention of being used as a decision-making tool at the time of a donor kidney offer. The risk index uses 13 donor-related parameters that would be known by the clinician at the time of the offer including age, height, weight and history of hypertension and diabetes.

The scientific and clinical practices underpinning the delivery of transplantation services have evolved over time. Further variation exists between different units and countries. As such, prediction models developed in a particular country may not be reliably applicable to populations in other countries in the future. It is therefore essential to externally validate proposed prediction models when considering their use in different populations and to revisit these validations over time [3–5].

We sought to validate the predictive performance of the KDRI in the UK kidney transplantation population. In our systematic review [6], we found that the KDRI has been validated in different populations across the globe [7–15]. In the UK, Watson et al. [14] assessed its performance in transplants performed between 2000 and 2007. The KDRI showed moderate discrimination in predicting the earliest of graft failure and death (C-index 0.63). The calibration has not previously been assessed in the UK kidney transplant population.

The survival process following transplantation consists of semi-competing events, where a terminal event precludes the observation of a non-terminal event, but not vice-versa. Specifically, in the context of kidney transplant survival outcomes, once a patient has died, we can no longer observe whether they experience graft failure. However, if a patient suffered graft failure, then we could still observe their death. In the existing literature on prediction models for graft failure, death is often not treated as a competing event, rather graft failure is censored by death or they are combined to predict a composite event.

The original KDRI defined graft failure as the earliest of graft failure or death. Predicting a composite outcome assumes that predictors have the same effect on both outcomes of interest [16], and in doing so, researchers shift the attention from the primary clinical endpoint of the proposed prediction model to one that may not be of clinical interest. Censoring the primary event of interest by the competing event violates the assumption of non-informative censoring typically used in standard time-to-event methods and can lead to bias in the cumulative incidence estimator, such that the sum of the individual event estimators exceeds the estimator of the composite event [17, 18]. Recent work has also noted the importance of accounting for competing events in external validation studies as well as in model development [19, 20].

The aim of this study was to externally validate the KDRI in the UK kidney transplantation adult population. Additionally, we aimed to explore whether modelling death as a competing event, rather than censoring recipients at the time of death, influences the predictive performance of the KDRI. Furthermore, we assessed whether updating the KDRI was required to improve predictions for graft failure.

## Methods

This study was reported in accordance with the Transparent reporting of a multivariable prediction model for Individual Prognosis or Diagnosis (TRIPOD) statement [21].

### Source of data

This was a cohort study based on a registry database collected by the UK Transplant Registry (UKTR) and held by NHS Blood and Transplant (NHSBT). Recipients were transplanted in the UK between January 1, 2004, and December 31, 2018. Recipients were followed-up until March 31, 2021.

### Participants

Adult recipients (aged 18 years and above) of a first, deceased donor, single, kidney-only transplant were included. Where recipients have had multiple transplants within the study period, only their first one was used for analysis. Recipients of en bloc, or multiple organ transplants (such as combined kidney and pancreas transplants), were not included.

### Outcomes

We assessed the performance of the KDRI for predicting graft failure 1 year and 5 years following kidney transplantation. Graft failure was defined as the time from transplantation until either return to dialysis or re-transplantation. In light of the semi-competing events, recipient death was handled in two ways: censoring patients at the time of death and modelling death as a competing event. The original KDRI was intended to predict graft failure; however, the model was developed to predict a composite outcome of time to the earliest of death or graft failure.

### Missing data

Recipients with both event time and indicator missing were excluded from the analysis. Missing values for donor height, weight, ethnicity, history of hypertension, history of diabetes, cause of death (cerebrovascular accident or not), creatinine value and hepatitis C virus status were imputed using multiple imputations with chained equations [22], assuming that the data were missing at random. None of the donors were missing age or type (deceased cardiac or deceased brain donor). Hence, these variables were not imputed but were included in the imputation model, along with the Aalen-Johansen estimates for the cumulative hazard. Continuous variables were imputed using predictive mean matching to ensure that implausible values were not imputed, such as negative values for height and weight.

12.78% of the patients had incomplete information for calculating the KDRI; therefore, we determined at least thirteen imputed data sets were required [23]. Fifteen imputed data sets were generated. For continuous variables, imputations were checked by comparing the distributions between imputed data sets. For binary and categorical variables, we checked whether the counts were similar between imputations (see Supplementary Material).

Parameter estimates and model performance measures, along with the associated standard errors, were pooled across the imputed data sets according to Rubin’s rules [24]. These pooled estimates and standard errors were used to construct 95% confidence intervals using the 97.5th quantile of the t-distribution.

### Sample size

The suitability of the sample size was determined according to the methods of Riley et al. [25]. While the sample size for this study was fixed (20,035 recipients), we also explored the mean standard error of the calibration slope for a range of sample sizes. These, along with further details on the sample size calculation, can be found in the Supplementary Material.

In the development article [2], the KDRI was split into quintiles, and Kaplan–Meier curves of the probability of graft survival were reported for each. We read the survival probabilities for the minimum and maximum quantiles and explored the sample size required for survival probabilities within that range. For 1-year graft failure, we considered survival probabilities 0.875, 0.901, 0.927 and 0.953, and for 5-year graft failure 0.635, 0.697, 0.760 and 0.822.

For 1- and 5-year graft failure, the mean standard error for the calibration slope varied between 0.053 and 0.092, and 0.036 and 0.051, respectively, for the survival probabilities under consideration (Table 1). We deemed these to be acceptable.

**Table 1:** Mean standard error of calibration slope from a simulation study of size 500, assuming a sample size of 20,000

Survival probability	Event time distribution rate	Mean calibration slope standard error
**1-year graft failure**		
0.875	0.118	0.053
0.901	0.092	0.061
0.927	0.067	0.074
0.953	0.042	0.092
**5-year graft failure**		
0.635	0.083	0.036
0.697	0.065	0.039
0.760	0.049	0.044
0.822	0.035	0.051

The linear predictor was assumed to follow Log-Norm(log(1.05), 0.42487)

### Summary statistics

The median time to graft failure was calculated using the Kaplan–Meier method, whereby the median time is given by the time at which the probability of survival is 0.5. Median follow-up times were calculated using the reverse Kaplan–Meier method. This is similar to the Kaplan–Meier method except the censoring indicator is treated as an event indicator.

### Model performance

#### Discrimination

Discrimination measures the rank separation between those who experience the outcome of interest and those who do not. For example, a model that discriminates well will predict a higher risk for a recipient that experiences graft failure than one who does not.

The discrimination was assessed using the time-dependent area under the receiver operating characteristic curve (AUC) [26]. Values typically range between 0.5 and 1, where 1 indicates perfect discrimination and 0.5 shows that predictions are as accurate as flipping a coin.

#### Calibration

Calibration is used to measure the agreement between observed and predicted risks. Here, we used the observed event proportion as a proxy for observed risk. As some patients were censored prior to the event time horizon, it was not possible to calculate the observed event proportion. To overcome this, we used a jack-knife approach to calculate pseudo-observations, which were then used as proxy measures of event indicators for censored patients [27].

Calibration plots using pseudo-observations with local weighted regression smoothing were assessed in each imputed dataset. For models that are well calibrated, the smoothed curve lies on the diagonal line that runs through the origin. Further, we calculated the calibration slope, where a value equal to one indicates perfect calibration. A calibration slope less than 1 suggests that predictions are too high for recipients with high event probabilities and too low for those with low probabilities. Conversely, a calibration slope greater than 1 suggests that recipients with high observed risk are under-estimated, and those with low observed risk are over-estimated. The calibration slope was calculated using a generalised linear model with pseudo-observations as the outcome and the complementary log–log transformed predicted risks as both an offset and covariate [27, 28]. The coefficient of the transformed risks indicates how far the calibration slope differs from 1; thus, the calibration slope is given by summing these two values.

Given that the baseline survival value for 1 and 5 years following transplantation was not reported in the original article, it was calculated within the UKTR data. Consequently, the calibration may appear more optimistic since the baseline cumulative incidence, which is required to calculate the absolute risk of graft failure, was estimated in the same cohort.

#### Overall prediction accuracy

The Brier score measures prediction error by estimating the squared difference between the event indicator and estimated risk [29]. Values closer to zero indicate a more accurate prediction model.

### Validation of the KDRI

The KDRI [2] can be calculated using

$$\mathrm{KDRI}=\exp\;\{-0.0194\mathrm I\left[\mathrm{age}<18\mathrm{yr}\right]\left(\mathrm{age}-18\mathrm{yr}\right)+0.0128\left(\mathrm{age}-40\right)+0.0107\mathrm I\left[\mathrm{age}>50\right]\left(\mathrm{age}-50\right)-\frac{0.0464\left(\mathrm{height}-170\right)}{10}-\frac{0.0199\mathrm I\left[\mathrm{weight}<80\right]\left(\mathrm{weight}-80\right)}5+0.1790\mathrm I\left[\mathrm{ethnicity}\;\mathrm{African}\;\mathrm{American}\right]+0.1260\mathrm I\left[\mathrm{history}\;\mathrm{of}\;\mathrm{hypertension}\right]+0.1300\mathrm I\left[\mathrm{history}\;\mathrm{of}\;\mathrm{diabetes}\right]+0.0881\mathrm I\left[\mathrm{cause}\;\mathrm{of}\;\mathrm{death}\;\mathrm{cerebrovascular}\;\mathrm{accident}\;\left(\mathrm{CVA}\right)\right]+0.2200\left(\mathrm{creatinine}-1\right)-0.2090\mathrm I\left[\mathrm{creatinine}>1.5\right]\left(\mathrm{creatinine}-1.5\right)+0.2400\mathrm I\left[\mathrm{Hepatitis}\;C\;\mathrm{virus}\left(\mathrm{HCV}\right)\;\mathrm{positive}\right]+0.1330\mathrm I\left[\mathrm{deceased}\;\mathrm{cardiac}\;\mathrm{donor}\;\left(\mathrm{DCD}\right)\right]\},$$

where $\mathrm{I}\left[.\right]$ is an indicator function which is equal to 1 if the criteria in [.] are satisfied and 0 otherwise. The KDRI originally derived by Rao et al. also considered transplant-related factors, such as cold ischaemic time, human leukocyte antigen mismatch and whether it was an en bloc or double kidney transplant. In practice, only the donor-related factors are used to calculate the KDRI [30]. With this in mind, we validated the donor-only KDRI. For each recipient, we calculated the linear predictor of the KDRI, by applying the natural logarithm to the index. Cox proportional hazards models [31] were used to assess the performance of the KDRI for predicting death-censored graft failure. In the presence of competing events, the Cox model can lead to biassed risk estimation. As alternatives, researchers typically use either the cause-specific Cox [32] or the Fine-Gray model [33]. The Fine-Gray model is often preferred when the goal is prediction rather than association [34]. However, in some instances, it is possible for the sum of patient-specific event probabilities, which should be constrained between zero and one, to exceed one [35]. Therefore, we used the cause-specific Cox models to validate the KDRI when accounting for death as a competing event.

### Updating the KDRI

To assess whether the KDRI required updating, we re-estimated the coefficients used in the original index. For the KDRI to be applicable in the UK cohort, we substituted African American ethnicity for Black ethnic origin. Variables were centred in the same way that they were in the original KDRI, and no further variable selection was undertaken. We re-estimated the coefficients by censoring graft failure at the time of death using a Cox proportional hazards model and accounting for death as a competing event using a cause-specific Cox model.

The coefficients were re-estimated in each of the 15 imputed datasets, and the performance of those updated models was individually assessed. The re-estimated coefficients and performance measures were then pooled according to Rubin’s rules.

When updating the KDRI, we assessed the predictive performance in the same group of recipients that were used to update the KDRI. This will naturally produce optimistic results. To account for optimism in the numerical summaries of predictive performance, we used Harrell’s bias correction method [36], with 100 bootstrap samples. Calibration plots were not adjusted for optimism thus represent the apparent calibration.

### Software

Multiple imputation was performed using Stata/MP 16.1 [37]. All other analyses were conducted in R 4.1.2 [38].

## Results

### Summary statistics

**Fig. 1:**
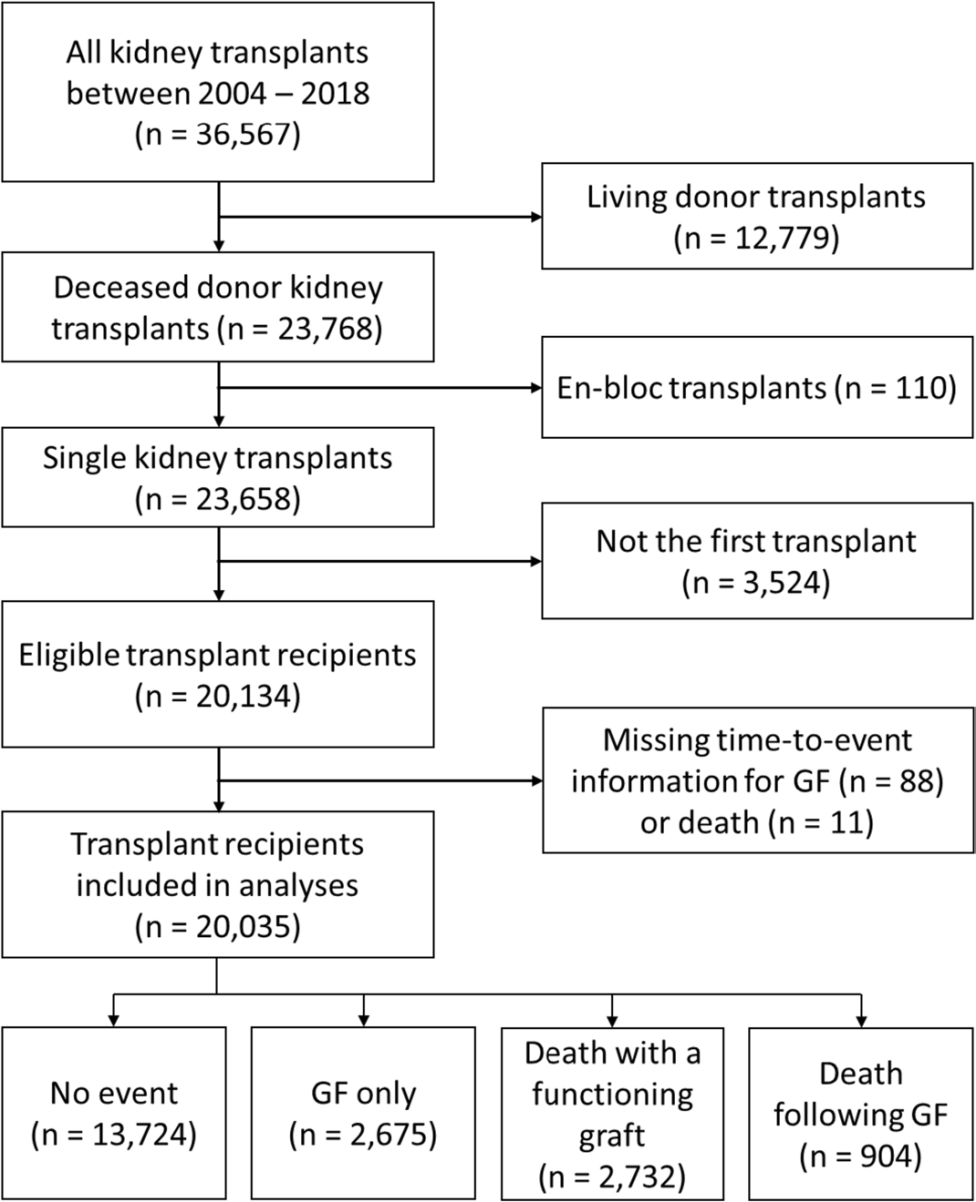
Flowchart of eligible transplant recipients for inclusion in analyses. GF graft failure

In total 20,134 deceased donor single kidney-only recipients who received a transplant between January 1, 2004, and December 31, 2018, in the UK were eligible for inclusion (Fig. 1). Eleven of the recipients had missing time-to-event information for both graft failure and death, and 88 missing for graft failure only. Therefore 20,035 transplants were included in our analysis.

The end of the follow-up period was March 31, 2021. The median follow-up time was 5.96 years, and the maximum follow-up time was 17.05 years. The minimum probability of survival for graft failure and for death was both above 0.5; hence, we did not observe the median survival time for either outcome in this study. At the end of the follow-up period, 13,724 (68.50%) recipients were alive with a functioning graft. 2675 (13.35%) recipients experienced graft failure only, and 904 (4.51%) died following graft failure. 2732 (13.64%) recipients died with a functioning graft (Fig. 1).

By the end of the first year following transplantation 1050 (5.24%) recipients had experienced graft failure only, and 186 (0.93%) died following graft failure. A total of 497 (2.48%) recipients had died with a functioning graft. By 5 years, 1936 (9.66%) recipients experienced graft failure alone, and 456 (2.28%) died following graft failure. 1509 (7.53%) transplant recipients died with a functioning graft.

A summary of the donor characteristics used to calculate the KDRI, including the number of missing values, is presented in Table 2, and a summary of recipient characteristics can be found in the Supplementary Material. Donors were aged between 1 and 85 years old, where 626 (3.13%) were younger than 18 years of age and 10,925 (54.53%) were older than 50. 10,646 (53.14%) donors weighed less than 80 kg. Creatinine was greater than 1.5 mg/dl for 1720 (8.59%) donors.

**Table 2:** Characteristics of donor patients in the UK kidney transplant population between January 1, 2004, and December 31, 2018

Variable	Mean [SD] or *N* (%)	Missing (%)
**Age, years**	49.91 [15.44]	0 (0)
**Height, cm**	170.29 [10.80]	269 (1.34)
**Weight, kg**	77.96 [17.77]	130 (0.65)
**Ethnicity**		63 (0.31)
Asian	372 (1.86)	
Black	222 (1.11)	
Chinese/oriental	56 (0.28)	
Mixed	148 (0.74)	
Other	160 (0.80)	
White	19,014 (94.90)	
**History of hypertension**		716 (3.57)
Yes	5296 (26.43)	
No	14,023 (69.99)	
**History of diabetes**		527 (2.63)
Yes	1275 (6.36)	
No	18,233 (91.01)	
**Cause of death**		161 (0.80)
CVA	741 (3.70)	
Not CVA	19,133 (95.50)	
**Creatinine, mg/dl**	0.95 [0.60]	1688 (8.43)
**HCV test result**		63 (0.32)
Positive	21 (0.10)	
Negative	19,951 (99.58)	
**Donor type**		0 (0)
DCD	7517 (37.52)	
DBD	12,518 (62.48)	

Numerical summaries of variables used to calculate the Kidney Donor Risk Index, including the number (and percentage) of missing values

*CVA* cerebrovascular accident, *HCV* hepatitis C virus, *DCD* deceased cardiac donor, *DBD* deceased brain donor

No values were missing for donor age and type of donor. 1688 (8.43%) donors had missing values for creatinine, the most of any variables required for calculating the KDRI. 2560 (12.78%) were missing at least one value required to calculate the KDRI.

**Fig. 2:**
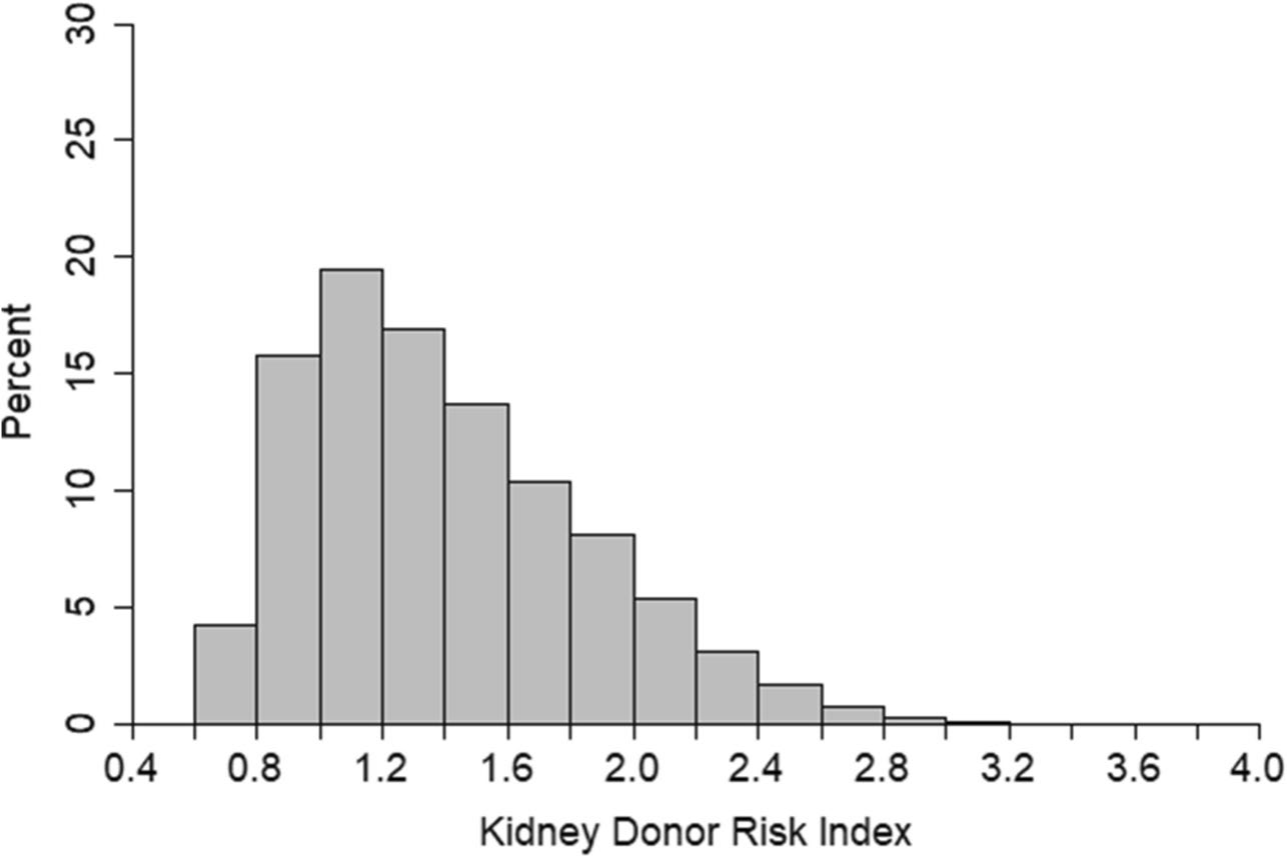
Distribution of the original Kidney Donor Risk Index in UK kidney transplantation population

The distribution of the KDRI in the original article by Rao et al. [2] was similar in shape to that of the transplants included in this analysis (Fig. 2). However, median KDRI values were higher in the UK cohort; 1.32 compared with 1.05 in the US cohort used to develop the index.

### External validation of the KDRI

#### One-year graft failure

The KDRI discriminated moderately well for predicting 1-year graft failure. The time-dependent AUC was 0.607 (95% CI 0.589 to 0.625) and 0.610 (95% CI 0.592 to 0.628) with and without accounting for competing events, respectively (Table 3).

**Table 3:** Numerical summary of the performance of the original Kidney Donor Risk Index for predicting graft failure 1 year and 5 years following transplantation while censoring for death and modelling death as a competing event

	Censoring at the time of death	Accounting for death as a competing event
**One-year graft failure**		
T-D AUC (95% CI)	0.610 (0.592, 0.628)	0.607 (0.589, 0.625)
Calibration slope (95% CI)	1.074 (0.878, 1.271)	1.074 (0.877, 1.272)
Brier Score (95% CI)	0.058 (0.055, 0.062)	0.058 (0.054, 0.061)
**Five-year graft failure**		
T-D AUC (95% CI)	0.625 (0.611, 0.640)	0.611 (0.597, 0.625)
Calibration slope (95% CI)	0.964 (0.827, 1.100)	0.979 (0.835, 1.123)
Brier Score (95% CI)	0.117 (0.112, 0.121)	0.114 (0.109, 0.118)

*T-D AUC* time-dependent area under receiver operating curve, *CI* confidence interval

**Fig. 3:**
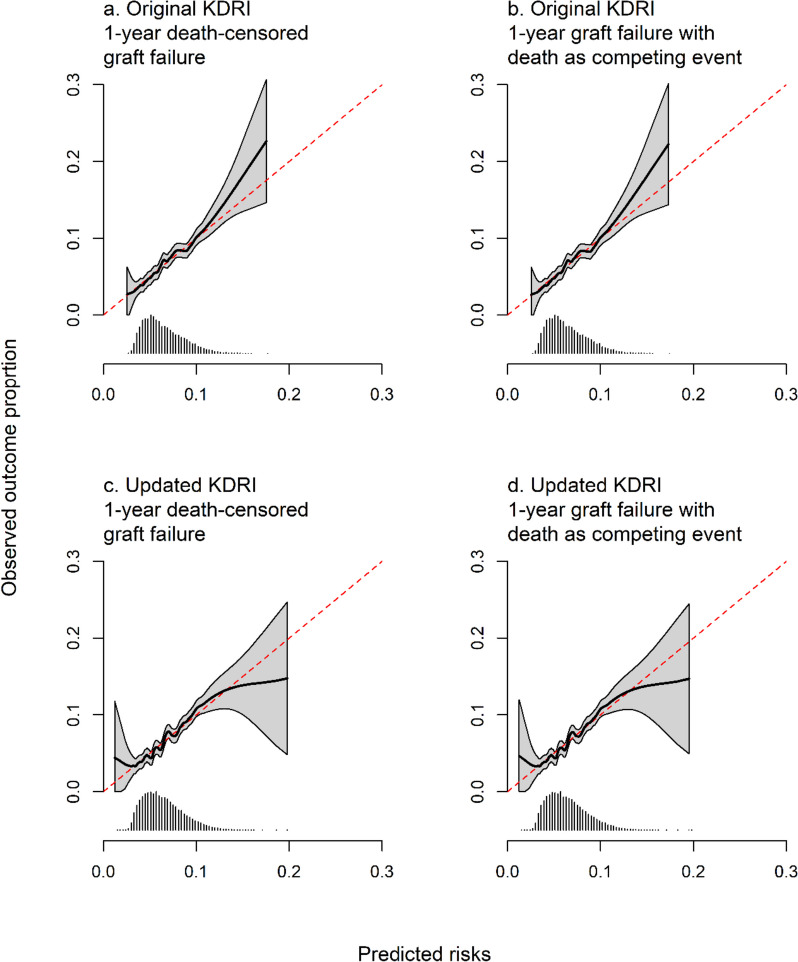
Calibration plots for the original and updated KDRI for predicting 1-year graft failure. The left panels show graft failure censoring at the time of death, and the right panels treat death as a competing event. Below each plot is a histogram of predicted risks. The dashed red line indicates perfect calibration

Calibration plots and slopes were similar when modelling graft failure while censoring for death and death as a competing event (Fig. 3a, b, Table 3). The KDRI was well calibrated for recipients with predicted risks less than 10%, but calibration was worse for those with predicted risks above this value. Only 1100 (5.5%) were at a higher risk than 10%, and for those, the KDRI underestimated the risk of graft failure. Calibration slopes were, respectively, 1.074 (95% CI 0.878 to 1.271) and 1.074 (95% CI 0.877 to 1.272) for predicting death-censored and competing event graft failure.

Predictive accuracy was the same regardless of whether death was handled as a competing event or not with reported Brier scores equal to 0.058 for both types of outcomes.

#### Five-year graft failure

Five years following kidney transplantation the time-dependent AUC was slightly lower when predicting graft failure with death as a competing event (0.611, 95% CI 0.597 to 0.625) as opposed to censoring at the time of death (0.625, 95% CI 0.611 to 0.640).

**Fig. 4:**
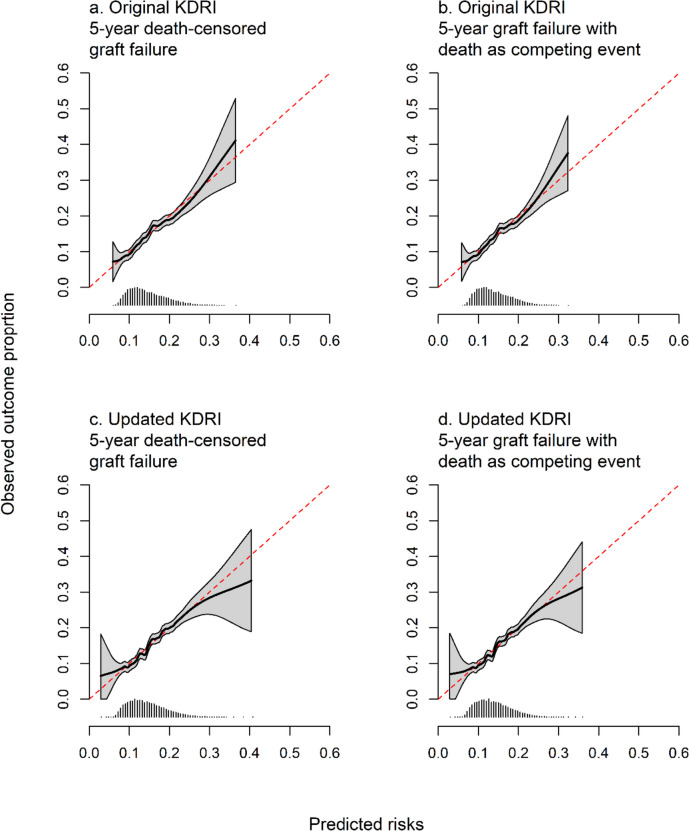
Calibration plots for the original and updated KDRI for predicting 5-year graft failure. The left panels show graft failure censoring at the time of death, and the right panels treat death as a competing event. Below each plot is a histogram of predicted risks. The dashed red line indicates perfect calibration

Using calibration plots, predicted risks using the KDRI were generally similar to the observed proportion of recipients who experienced graft failure (Fig. 4a, b). Calibration was poorest for those at higher risk of graft failure. The risk of graft failure was underestimated for recipients at a higher risk. The calibration slopes were 0.964 (95% CI 0.827 to 1.100) when censoring recipients at the time of death and 0.979 (95% CI 0.835 to 1.123) when modelling death as a competing event.

Predictions were less accurate for 5 years compared with a 1-year graft failure. Brier scores differed for death-censored and competing event graft failure (0.117, 95% CI 0.112 to 0.121 and 0.114, 95% CI 0.109 to 0.118, respectively).

### Updating the KDRI

To update the KDRI, we re-estimated the coefficients used in the original index in the UK kidney transplant population. No additional predictors were considered. In the updated models, the estimates were similar regardless of whether the death was modelled as a competing event or not (Table 4). Confidence intervals for the effect of ethnicity were much wider than in the original index, likely because only 1.11% of donors were of Black ethnic origin.

**Table 4:** Coefficients (and 95% confidence intervals) of the variables used to calculate the Kidney Donor Risk Index from the original development, the updated Cox proportional hazards model and the updated cause-specific Cox model

Variable	Original	Updated (Cox model)	Updated (cause-specific Cox model)
Age-18; for donors under 18 years	− 0.019 (− 0.031, − 0.010)	− 0.032 (− 0.096, 0.033)	− 0.031 (− 0.095, 0.033)
Age-40, years	0.013 (0.011, 0.015)	0.016 (0.009, 0.024)	0.016 (0.009, 0.024)
Age-50; for donors over 50 years	0.011 (0.005, 0.016)	0.003 (− 0.010, 0.016)	0.003 (− 0.010, 0.016)
Height per 10 cm increase	− 0.046 (− 0.062, − 0.031)	− 0.034 (− 0.093, 0.026)	− 0.034 (− 0.093, 0.026)
Weight per 5 kg increase; for donors below 80 kg	− 0.020 (− 0.031, − 0.010)	− 0.035 (− 0.068, − 0.002)	− 0.035 (− 0.068, − 0.002)
Ethnicity			
Not Black ethnic origin	Reference		
Black ethnic origin	0.179 (0.122, 0.239)	0.409 (− 0.017, 0.835)	0.405 (− 0.021, 0.832)
History of hypertension			
No	Reference		
Yes	0.126 (0.077, 0.174)	0.256 (0.135, 0.378)	0.255 (0.133, 0.376)
History of diabetes			
No	Reference		
Yes	0.130 (0.039, 0.215)	0.154 (− 0.050, 0.358)	0.153 (− 0.051, 0.357)
Cause of death CVA			
No	Reference		
Yes	0.088 (0.039, 0.131)	− 0.035 (− 0.308, 0.238)	− 0.036 (− 0.309, 0.237)
Creatinine-1, mg/dl	0.220 (0.157, 0.285)	0.395 (0.205, 0.586)	0.395 (0.204, 0.586)
Creatinine-1; for donors with creatinine > 1.5, mg/dl	− 0.209 (− 0.301, − 0.117)	− 0.500 (− 0.819, − 0.182)	− 0.500 (− 0.818, − 0.181)
HCV			
Negative	Reference		
Positive	0.240 (0.122, 0.358)	0.233 (− 1.286, 1.751)	0.234 (− 1.284, 1.753)
Donor type			
DBD	Reference		
DCD	0.133 (0.020, 0.247)	0.113 (0.005, 0.221)	0.112 (0.004, 0.220)

Variables are centred as they were in the original publication

*CVA* cerebrovascular accident, *DBD* deceased brain donor, *DCD* deceased cardiac donor

The effect of age for those under 18 and over 50 years and height was not found to be associated with graft failure. Additionally, donor ethnicity and donor HCV status were not significantly associated with graft survival.

#### One-year graft failure

Discrimination was similar for predicting death-censored graft failure (time-dependent AUC 0.614) and graft failure with death as a competing event (time-dependent AUC 0.608) (Table 5).

**Table 5:** Numerical summary of the performance of the updated Kidney Donor Risk Index for predicting graft failure 1 year and 5 years following transplantation while censoring for death and modelling death as a competing event

	Censoring at the time of death	Accounting for death as a competing event
**One-year graft failure**		
T-D AUC (optimism)	0.614 (− 0.00015)	0.608 (0.00252)
Calibration slope (optimism)	1.096 (− 0.00049)	1.068 (0.02812)
Brier score (optimism)	0.058 (− 0.00004)	0.058 (− 0.00007)
**Five-year graft failure**		
T-D AUC (optimism)	0.629 (− 0.00019)	0.612 (0.00254)
Calibration slope (optimism)	1.016 (− 0.00133)	1.002 (0.02911)
Brier score (optimism)	0.117 (0.00006)	0.114 (− 0.00010)

*T-D AUC* time-dependent area under receiver operating curve

Calibration slopes were 1.096 when censoring at the time of death and 1.068 when modelling death as a competing event. Calibration plots were similar for both types of graft failure and clearly showed that low risks were over-estimated and high risks were under-estimated (Fig. 3c, d). There was no difference in prediction accuracy whether accounting for death as a competing event or not, with Brier scores equal to 0.058 for both cases.

#### Five-year graft failure

Discrimination was lower when modelling graft failure with death as a competing event (time-dependent AUC 0.629 and 0.612, respectively) for the updated index (Table 5).

We found calibration slopes were 1.016 for death-censored graft failure and 1.002 when modelling death as a competing event. The calibration plots (Fig. 4c, d) showed miscalibration for recipients at the highest and lowest predicted risks, and 95% confidence intervals were much wider at the tails of the curve.

Prediction accuracy was slightly improved when modelling death as a competing event compared to censoring at the time of death with Brier scores equal to 0.114 and 0.117, respectively.

## Discussion

### Principal findings

In external validation, the KDRI had moderate discrimination and was generally well calibrated for predicting graft failure 1 year and 5 years following kidney transplantation. For predicting 1-year graft failure discrimination, calibration and predictive accuracy did not differ depending on how death prior to graft failure was handled. Discrimination was higher for predicting 5-year graft failure. Predictions were more accurate for early graft failure compared to those at 5 years following transplantation.

Calibration slopes indicated miscalibration in the KDRI; however, the corresponding 95% confidence intervals were wide. In calibration plots for both outcomes, miscalibration was mainly driven by recipients at higher risk, where the event probabilities were generally underestimated. However, it should be noted that the baseline survival was not reported in the original article and as such has been estimated within the same cohort as it is being validated in. Therefore, the calibration of the KDRI in the UK kidney transplant population may be more optimistic.

Updating the KDRI in the UK kidney transplant population yielded similar coefficients, but some prognostic factors were no longer associated with graft failure. Given that the coefficient estimates did not differ between the Cox and the cause-specific Cox models, it is unsurprising that there was little difference between the predictive performance of those models.

### Strengths and limitations

To our knowledge, this is the first study to assess the performance of the KDRI under a semi-competing risks framework for first, deceased donor, single, kidney-only and adult transplants. Our work included all eligible kidney transplants that occurred in the UK during the study period, with a long follow-up period. The KDRI was previously validated in the UK by Watson et al. [14] using information on kidney transplants that occurred between 2000 and 2007. Therefore, our work serves to assess whether the KDRI is still relevant in the UK kidney transplant population.

The baseline survivor function was recalibrated for our cohort; therefore, calibration may seem more optimistic in this external validation of the KDRI. From the current analyses, we cannot comment on the clinical utility of the index in the UK kidney transplant population. Further work is required to determine whether the KDRI is clinically relevant in practice.

Few recipients experienced a competing event (died with a functioning graft), which may explain why little difference was found in predictive performance when considering death as a competing event and censoring graft failure at the time of death. There is a lack of guidance concerning under what situation ignoring the competing risk elements can impact the performance of the prediction models. Externally validating and updating a model under the competing risk framework serves as a sensitivity analysis to evaluate the developed models which ignore the competing risk elements. Future work could explore to what extent the proportion of non-terminal events censored by the terminal events impacts the predictive performance. This can potentially lead to recommendations for practice for when it is necessary to account for competing events and when traditional methods, such as the Cox proportional hazards model, might suffice.

### Results in context

The KDRI only considers donor-related variables to predict graft failure in the recipient of the kidney transplant. Additional donor variables may improve predictive performance. The Maryland Aggregate Pathology Index (MAPI) [39], for example, utilises information gathered from biopsies of donor kidneys and has shown higher discrimination in internal and external validation [9, 40]. Such additional information may be able to improve performance. However, it may not be practical in a decision-making tool since, in the UK, this information may not be known at the time of the offer of a donor kidney. Additionally, utilising information about the recipient and the transplant process, or other existing indices which incorporate these variables, could also improve predictions.

A validation study in the US [9] evaluated the predictive performance of the KDRI 2 years following transplantation and showed poor discrimination with time-dependent AUC equal to 0.45. External validation in Australia and New Zealand [15] reported C-index 0.63 (95% CI 0.60 to 0.65) for predicting death-censored graft failure. In Canada, the KDRI showed moderate discrimination with C-index equal to 0.59 [13]. The KDRI was previously validated using data from kidney transplants performed in the UK between 2000 and 2007 [14] and reported a C-index of 0.63.

Zhong et al. [41] also assessed whether the original KDRI required updating using information on kidney transplants performed between 2000 and 2016 in the US. Their updated index showed marginally higher discrimination than the original KDRI (original KDRI C-index 0.651; updated KDRI C-index 0.652); however, the calibration was not assessed. This study also determined that there is little to be gained in updating the KDRI.

## Conclusions

The Kidney Donor Risk Index, originally developed in the US population, showed moderate predictive performance overall in our external validation in the UK kidney transplant population. The use of a semi-competing risks framework made a slight difference when predicting 5-year graft failure compared to censoring for death. The updated index had slightly improved discrimination but was poorly calibrated for those with the highest and lowest risk of graft failure. Therefore, we conclude that updating the KDRI in the present form is not required.

### Supplementary Information

**Additional file 1: Fig. S1.** Imputed datasets: Summary of continuous variables. **Tables S1.** Imputed datasets: Summary of categorical variables. **Table S2.** Sample size calculations. **Table S3.** Summary of recipient and transplant related factors.

## Data Availability

The data underlying this article can be requested from the data controller, NHS Blood and Transplant. The code used to conduct these analyses is available from GitHub: https://github.com/Yinghui-Wei-team/kdrivalidation.
