# Supplementary material for: An external validation of the Kidney Donor Risk Index in the UK transplant population in the presence of semi-competing events
Source: Diagn Progn Res. 2023 Nov 21;7:20. doi: 10.1186/s41512-023-00159-9 (PMC10662562; doi:10.1186/s41512-023-00159-9)
Supplement: Supplementary file 1 — Additional file 1: Fig. S1. Imputed datasets: Summary of continuous variables. Tables S1. Imputed datasets: Summary of categorical variables. Table S2. Sample size calculations. Table S3. Summary of recipient and transplant related factors. [file 41512_2023_159_MOESM1_ESM.pdf]

Supplementary material

Missing data

The following plots show the distribution of donor creatinine, height, KDRI, and weight in the original dataset (indicated by 0) and each imputed dataset (1-15).

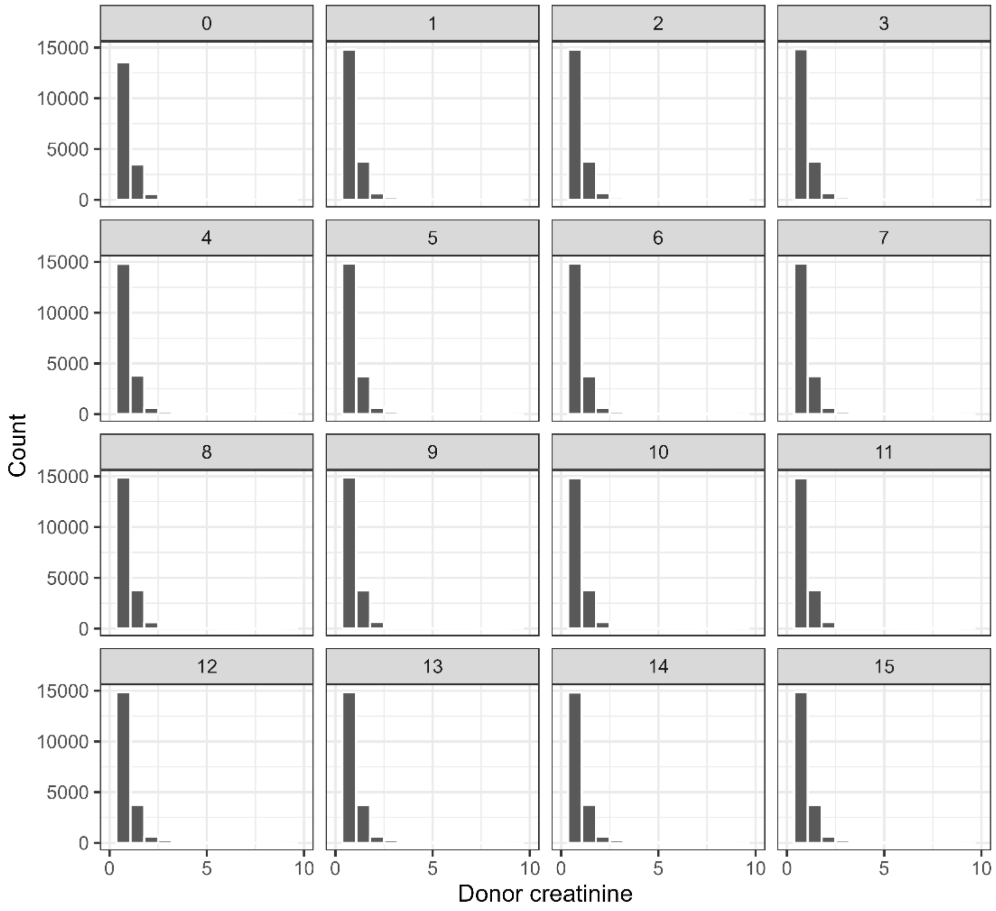

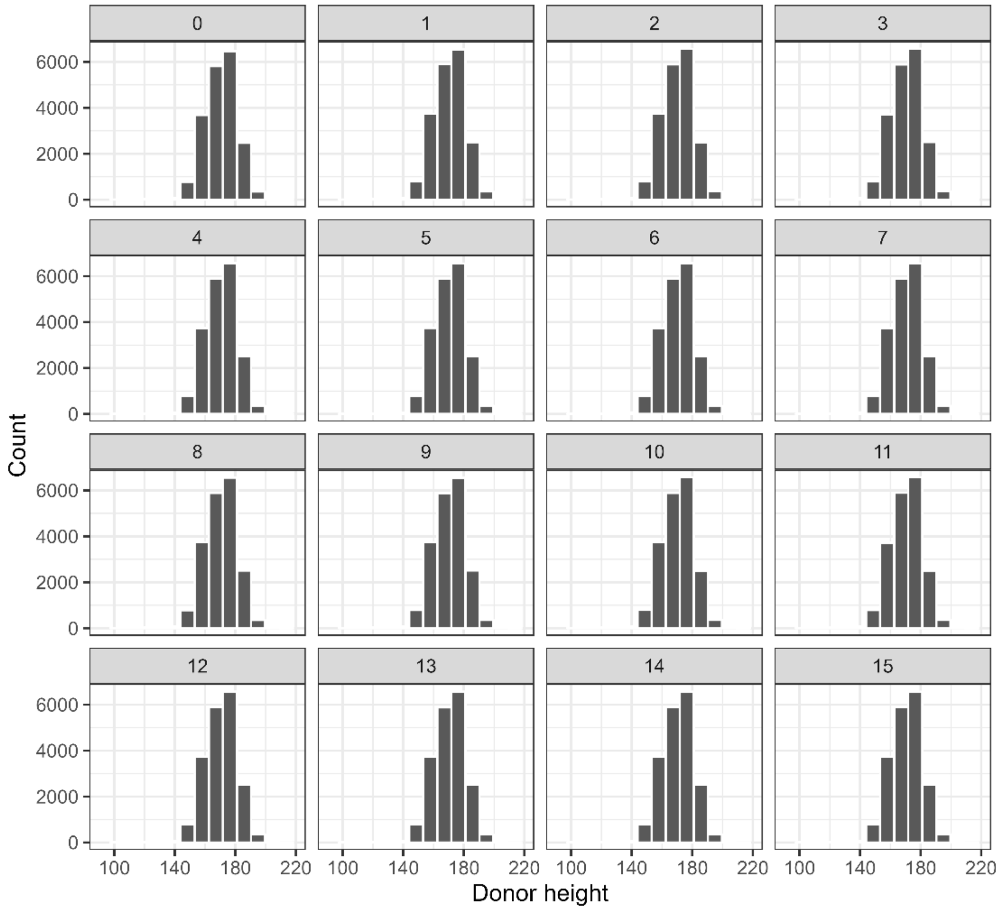
0
1
2
3
4
5
6
7
8
9
10
11
12
13
14
15
Count
6000
4000
2000
0
100
140
180
220
Donor height

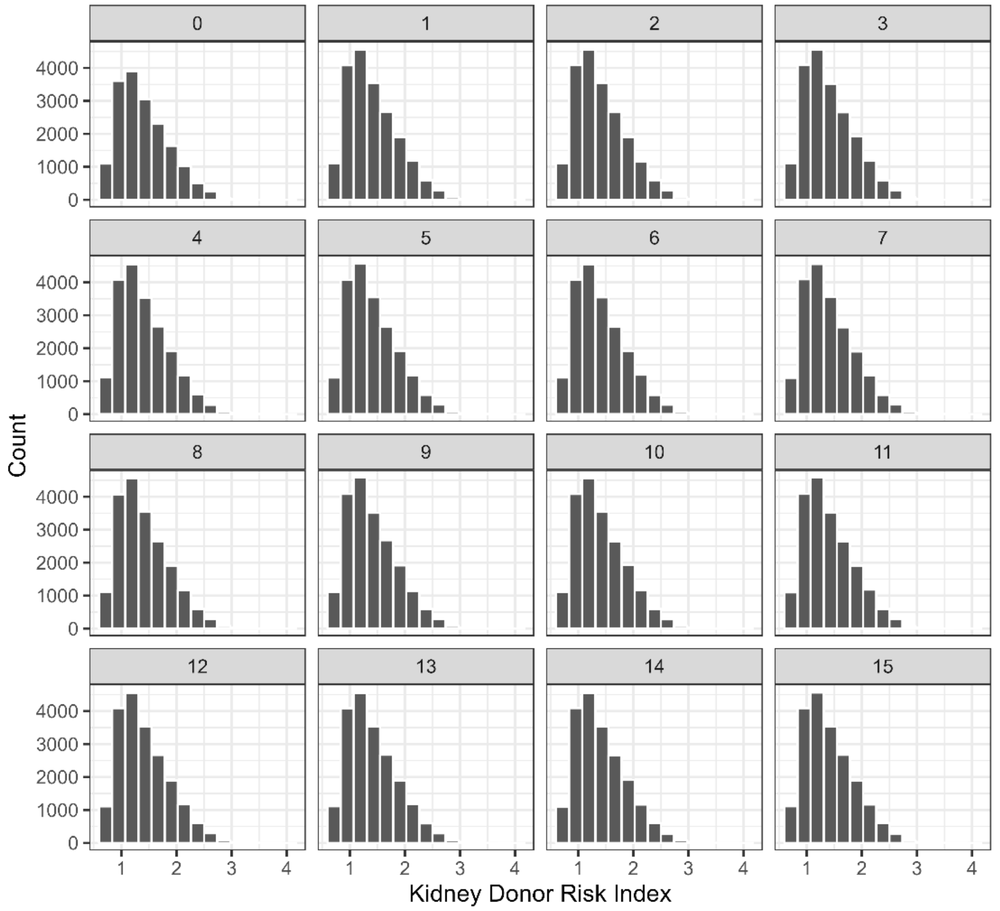
0
1
2
3
4
5
6
7
8
9
10
11
12
13
14
15
Count
4000
3000
2000
1000
0
1
2
3
4
Kidney Donor Risk Index

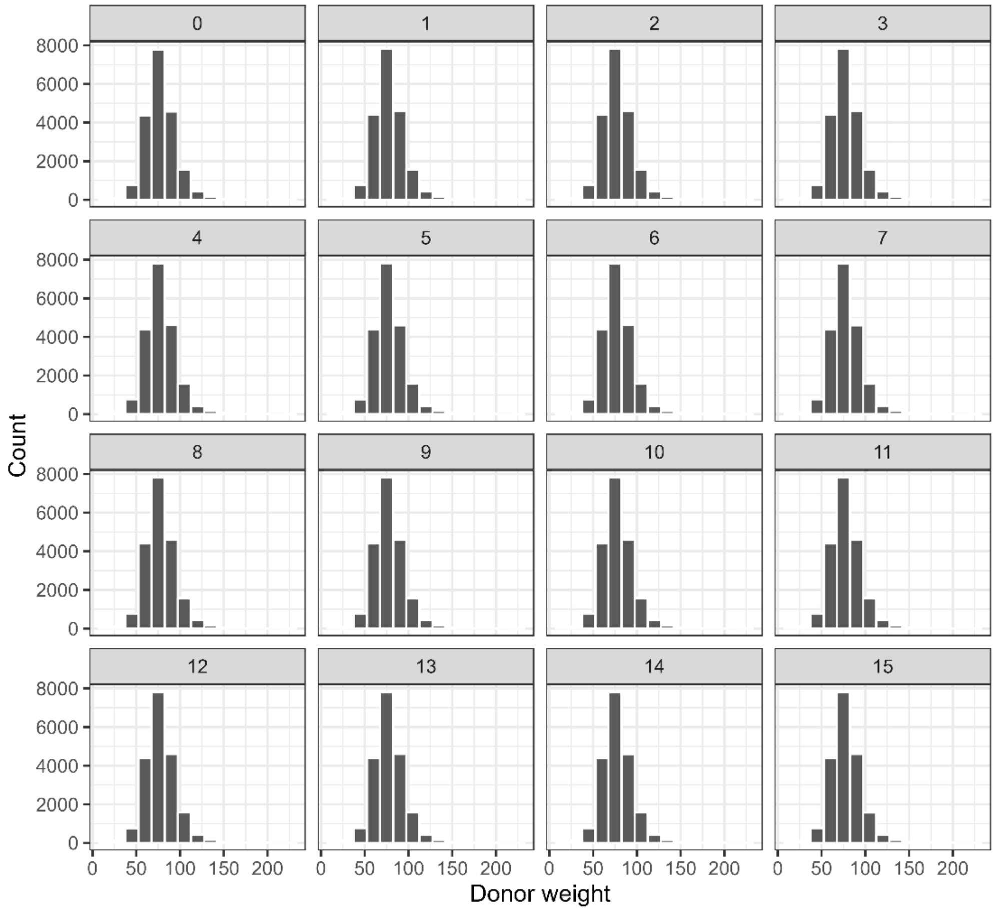

0
1
2
3
4
5
6
7
8
9
10
11
12
13
14
15
8000
6000
4000
2000
0
Count
0
50
100
150
200
Donor weight

The table below shows the number and percentage of categorical variables ethnicity, history of hypertension, history of diabetes, cause of death, and HCV status in the original dataset and each imputed dataset (1-15).

**Characteristic**	**Original**	**1**	**2**	**3**	**4**	**5**	**6**	**7**	**8**	**9**	**10**	**11**	**12**	**13**	**14**	**15**
Ethnicity																
Black ethnic origin	222 (1.11%)	222 (1.11%)	222 (1.11%)	224 (1.12%)	223 (1.11%)	223 (1.11%)	224 (1.12%)	222 (1.11%)	222 (1.11%)	224 (1.12%)	223 (1.11%)	223 (1.11%)	222 (1.11%)	222 (1.11%)	223 (1.11%)	222 (1.11%)
Not Black ethnic origin	19,750 (98.89%)	19,813 (98.89%)	19,813 (98.89%)	19,811 (98.88%)	19,812 (98.89%)	19,812 (98.89%)	19,811 (98.88%)	19,813 (98.89%)	19,813 (98.89%)	19,811 (98.88%)	19,812 (98.89%)	19,812 (98.89%)	19,813 (98.89%)	19,813 (98.89%)	19,812 (98.89%)	19,813 (98.89%)
History of hypertension	5,296 (27.41%)	5,467 (27.29%)	5,483 (27.37%)	5,503 (27.47%)	5,477 (27.34%)	5,484 (27.37%)	5,485 (27.38%)	5,477 (27.34%)	5,475 (27.33%)	5,483 (27.37%)	5,499 (27.45%)	5,487 (27.39%)	5,468 (27.29%)	5,480 (27.35%)	5,484 (27.37%)	5,468 (27.29%)
History of diabetes	1,275 (6.54%)	1,320 (6.59%)	1,309 (6.53%)	1,308 (6.53%)	1,315 (6.56%)	1,317 (6.57%)	1,307 (6.52%)	1,301 (6.49%)	1,307 (6.52%)	1,310 (6.54%)	1,312 (6.55%)	1,314 (6.56%)	1,307 (6.52%)	1,323 (6.60%)	1,311 (6.54%)	1,315 (6.56%)
Cause of death																
CVA	741 (3.73%)	748 (3.73%)	754 (3.76%)	745 (3.72%)	748 (3.73%)	746 (3.72%)	745 (3.72%)	747 (3.73%)	744 (3.71%)	748 (3.73%)	745 (3.72%)	748 (3.73%)	746 (3.72%)	749 (3.74%)	748 (3.73%)	749 (3.74%)
Not CVA	19,133 (96.27%)	19,287 (96.27%)	19,281 (96.24%)	19,290 (96.28%)	19,287 (96.27%)	19,289 (96.28%)	19,290 (96.28%)	19,288 (96.27%)	19,291 (96.29%)	19,287 (96.27%)	19,290 (96.28%)	19,287 (96.27%)	19,289 (96.28%)	19,286 (96.26%)	19,287 (96.27%)	19,286 (96.26%)
HCV status																
Negative	19,951 (99.89%)	20,014 (99.90%)	20,013 (99.89%)	20,014 (99.90%)	20,014 (99.90%)	20,014 (99.90%)	20,014 (99.90%)	20,014 (99.90%)	20,014 (99.90%)	20,014 (99.90%)	20,014 (99.90%)	20,014 (99.90%)	20,014 (99.90%)	20,014 (99.90%)	20,014 (99.90%)	20,014 (99.90%)
Positive	21 (0.11%)	21 (0.10%)	22 (0.11%)	21 (0.10%)	21 (0.10%)	21 (0.10%)	21 (0.10%)	21 (0.10%)	21 (0.10%)	21 (0.10%)	21 (0.10%)	21 (0.10%)	21 (0.10%)	21 (0.10%)	21 (0.10%)	21 (0.10%)

CVA: cerebrovascular accident; HCV: hepatitis C virus.

Note: The percentage in the column for the original (incomplete) data represents the percentage of available cases.

Sample size calculation

Using the information reported in the development of the KDRI, we assumed that KDRI ~ Log-Normal(log(1.05), 0. 42487). The original publication reported C-statistic (0.62), which was then converted to Royston's D to calculate the standard error of the linear predictor. The simulation was of size 500.

We assumed that the survival times originated from and Exponential distribution for a range of rates, to reflect the range of survival probabilities of interest. 72% of patients in the development cohort were alive with a functioning graft at the end of follow-up, thus we aimed to simulate patient data such that 72% were censored. The censoring distribution was also assumed to be Exponential, with the rate estimated using the method of Wan [S1]. The maximum follow-up was ten years to mimic the development cohort.

Survival probability	Rate	Sample size	Calibration slope SE
**1-year graft failure**			
0.875	0.118	19000	0.055
		19500	0.054
		20000	0.053
		20500	0.053
		21000	0.052
0.901	0.092	19000	0.063
		19500	0.062
		20000	0.061
		20500	0.060
		21000	0.060
0.927	0.067	19000	0.076
		19500	0.075
		20000	0.074
		20500	0.073
		21000	0.072
0.953	0.042	19000	0.094
		19500	0.092
		20000	0.092
		20500	0.091
		21000	0.089
**5-year graft failure**			
0.635	0.083	19000	0.037
		19500	0.036
		20000	0.036
		20500	0.035
		21000	0.035
0.697	0.065	19000	0.040
		19500	0.040
		20000	0.039
		20500	0.038
		21000	0.038

0.760	0.049	19000	0.045
		19500	0.044
		20000	0.044
		20500	0.043
		21000	0.043
0.822	0.035	19000	0.052
		19500	0.051
		20000	0.051
		20500	0.050
		21000	0.050

References

[S1] Wan F. Simulating survival data with predefined censoring rates for proportional hazards models. Statistics in Medicine. 2017;36(5):838-54.

Summary statistics

Summary of recipient and transplant related factors.

Variable	Mean [SD] or N (%)	Missing (%)
**Age, years**	51.40 [13.38]	0 (0)
**Height, cm**	169.53 [10.57]	5,284 (26.37)
**Weight, kg**	76.84 [16.55]	1,075 (5.37)
**Ethnicity**		128 (0.64)
Asian	2,957 (14.76)	
Black	1,585 (7.91)	
Chinese/Oriental	245 (1.22)	
Mixed	63 (0.31)	
Other	305 (1.52)	
White	14,752 (73.63)	
**Diabetes**		0 (0)
Yes	1,945 (9.71)	
No	18,090 (90.29)	
**CIT, hours**	15.29 [5.53]	174 (0.87)
**HLA match group**		2 (<0.01)
1	2,187 (10.92)	
2	6,667 (33.28)	
3	9,725 (48.54)	
4	1,454 (7.26)	
**HLA match grade**		2 (<0.01)
Zero mismatch	2,187 (10.92)	
Favourable match	4,473 (22.33)	
Non-favourable match	13,373 (66.75)	

CIT: cold ischaemic time; HLA: human leukocyte antigen
